# Health-related quality of life and symptoms in patients with IPF treated with nintedanib: analyses of patient-reported outcomes from the INPULSIS® trials

**DOI:** 10.1186/s12931-020-1298-1

**Published:** 2020-01-30

**Authors:** Michael Kreuter, Wim A. Wuyts, Marlies Wijsenbeek, Sabrina Bajwah, Toby M. Maher, Susanne Stowasser, Natalia Male, Wibke Stansen, Nils Schoof, Leticia Orsatti, Jeffrey Swigris

**Affiliations:** 1 Center for Interstitial and Rare Lung Diseases, Pneumology, Thoraxklinik, Heidelberg University Hospital, Röntgenstraße 1, 69126 Heidelberg, Germany; 2 German Center for Lung Research, Gießen, Germany; 3 Unit for Interstitial Lung Diseases, Department of Pulmonary Medicine, University Hospitals Leuven, Leuven, Belgium; 4 Erasmus University, Rotterdam, the Netherlands; 5 King’s College London, London, UK; 6 National Heart and Lung Institute, Imperial College, London, UK; 7 Interstitial Lung Disease Unit, Royal Brompton Hospital, London, UK; 8 Boehringer Ingelheim International GmbH, Ingelheim am Rhein, Germany; 9 Boehringer Ingelheim GmbH & Co KG, Ingelheim am Rhein, Germany; 10 University of Colorado, Denver, CO USA

**Keywords:** SGRQ, UCSD-SOBQ, CASA-Q, EQ-5D VAS

## Abstract

**Background:**

In the Phase III INPULSIS® trials, treatment of patients with idiopathic pulmonary fibrosis (IPF) with nintedanib significantly reduced the annual rate of decline in forced vital capacity (FVC) versus placebo, consistent with slowing disease progression. However, nintedanib was not associated with a benefit in health-related quality of life (HRQoL) assessed using the St George’s respiratory questionnaire (SGRQ). We aimed to further examine the impact of IPF progression on HRQoL and symptoms, and to explore the effect of nintedanib on HRQoL in patients from the INPULSIS® trials stratified by clinical factors associated with disease progression.

**Methods:**

Patient-reported outcome (PRO) data from the INPULSIS® trials were included in three *post hoc* analyses. Two analyses used the pooled data set to examine PRO changes from baseline to week 52 according to 1) decline in FVC and 2) occurrence of acute exacerbations. In the third analysis, patients were stratified based on clinical indicators of disease progression (gender, age and physiology [GAP] stage; FVC % predicted; diffusing capacity of the lung for carbon monoxide [DL_CO_] % predicted; composite physiologic index [CPI]; and SGRQ total score) at baseline; median change from baseline was measured at 52 weeks and treatment groups were compared using the Wilcoxon two-sample test.

**Results:**

Data from 1061 patients (638 nintedanib, 423 placebo) were analyzed. Greater categorical decline from baseline in FVC % predicted over 52 weeks was associated with significant worsening of HRQoL and symptoms across all PRO measures. Acute exacerbations were associated with deterioration in HRQoL and worsened symptoms. In general, patients with advanced disease at baseline (defined as GAP II/III, FVC ≤ 80%, DL_CO_ ≤ 40%, CPI >  45, or SGRQ > 40) experienced greater deterioration in PROs than patients with less-advanced disease. Among patients with advanced disease, compared with placebo, nintedanib slowed deterioration in several PROs; benefit was most apparent on the SGRQ (total and activity scores).

**Conclusions:**

In patients with advanced IPF, compared with placebo, nintedanib slowed deterioration in HRQoL and symptoms as assessed by several PROs. HRQoL measures have a higher responsiveness to change in advanced disease and may lack sensitivity to capture change in patients with less-advanced IPF.

## Background

Idiopathic pulmonary fibrosis (IPF) is a chronic, progressive and fibrotic lung disease characterized by declining pulmonary function leading to respiratory failure and death [1]. Based on data collected prior to the availability of antifibrotic therapy, post-diagnosis survival for patients with IPF is typically 3–5 years [2, 3]. As IPF worsens, the development of comorbidities, increased symptom burden and the need for long-term oxygen therapy contribute to increasing impairments in health-related quality of life (HRQoL) [4, 5]. Patient-reported outcomes (PROs) can quantify how a disease may affect a patient’s HRQoL, and a wide range of PRO measures have been employed to understand the impact of HRQoL in IPF [6].

In patients with advanced IPF, as lung function declines, HRQoL follows. Registry studies reveal that a low forced vital capacity (FVC) % predicted (ie ≤ 50) is associated with severe symptoms and poor HRQoL, and a significant decline in FVC is associated with worsening symptoms and deterioration in HRQoL [4, 7]. Furthermore, as patients with IPF approach acute exacerbation or death, they experience an escalating symptom burden and a rapid decline in HRQoL [5, 8].

In the Phase III INPULSIS® trials, treatment with nintedanib significantly reduced the annual rate of decline in FVC in patients with IPF, compared with placebo, reflecting a slowing of disease progression [9]. Additionally, in a prespecified pooled analysis, treatment with nintedanib significantly increased the time to first adjudicated, confirmed or suspected, acute exacerbation of IPF [9]. However, nintedanib was not associated with a significant treatment difference in HRQoL as assessed using the St George’s respiratory questionnaire (SGRQ) [9], a PRO that has demonstrated acceptable psychometric properties in IPF [10, 11].

Using data gathered in the INPULSIS® trials, we conducted three *post hoc* analyses to examine the impact of the following on HRQoL and symptoms in patients with IPF: 1) decline in FVC % predicted; 2) occurrence of acute exacerbations; and 3) treatment with nintedanib.

## Methods

### INPULSIS® studies

The two INPULSIS® studies were 52-week, randomized, double-blind, placebo-controlled, parallel-group Phase III trials, in which patients with IPF received either placebo or nintedanib 150 mg twice daily [9]. Enrolled patients had an FVC % predicted ≥ 50% and a diffusing capacity of the lung for carbon monoxide (DL_CO_) % predicted of 30–79%.

The primary outcome measure in the INPULSIS® trials was the annual rate of decline in FVC [9]. Time to first acute exacerbation and change from baseline in SGRQ total score were secondary outcome measures. The SGRQ comprises three domains (symptoms, activity and impacts); the total score and the score for each domain range from 0 to 100, with higher scores indicating worse HRQoL [10, 12]. The minimal important difference (MID) for each domain is: 8 (symptoms), 5 (activity) and 7 (impacts and total) [10]. Several other PROs were used in the INPULSIS® trials: the University of California San Diego shortness of breath questionnaire (UCSD-SOBQ); the Cough and Sputum Assessment Questionnaire (CASA-Q); and the EuroQoL 5-dimensional quality of life questionnaire (EQ-5D) visual analog scale (VAS). The UCSD-SOBQ total score ranges from 0 to 120, with higher scores indicating greater severity of breathlessness; the MID is estimated to be 8 (range 5–11) [13]. The CASA-Q comprises four domains (symptom and impact for cough and sputum); the score for each domain ranges from 0 to 100, and lower scores indicate worse symptoms / greater impact [14]. Data support the validity of the UCSD-SOBQ and the CASA-Q for assessing target domains in patients with IPF [15]. The EQ-5D is a generic measure that has been used to assess HRQoL in IPF [5]. The VAS of the EQ-5D is scored between 0 and 100, with lower values indicating worse health [16].

### Data analyses

Three *post hoc* analyses of the pooled dataset from the INPULSIS® trials were conducted using HRQoL and symptom data from the PRO measures described above. For each PRO, median change from baseline at 52 weeks was evaluated. Patients with a missing FVC value at week 52 were excluded. Patients with missing values for a PRO at baseline and/or week 52 were excluded from the analysis of that PRO. For analysis 1 (see below), sensitivity analyses were conducted that included patients with imputation of missing values (last observation carried forward [LOCF] or worst observation carried forward [WOCF]).

### Analysis 1: impact of decline in FVC on HRQoL and symptoms

The objective of this analysis was to evaluate change in HRQoL and symptoms from baseline to week 52 in three subgroups defined by absolute decline in FVC % predicted over the study period.

Previously published data show that absolute declines in FVC % predicted of ≥ 5% or ≥ 10% are associated with mortality, and the estimated minimal clinically important difference for FVC % predicted is 2–6% [17, 18]. We stratified the sample thus: ≤ 5% decline in FVC % predicted; > 5–≤ 10% decline in FVC % predicted; > 10% decline in FVC % predicted. Data from nintedanib- and placebo-treated patients were pooled for this analysis. Between-groups comparisons were made using two-group Satterthwaite t-tests.

### Analysis 2: impact on HRQoL and symptoms of acute exacerbations

The objective of this analysis was to compare changes in HRQoL and symptoms from baseline to week 52 between patients who experienced investigator-reported acute exacerbations and those who did not. Patients were stratified into two groups: 1) those who experienced at least one investigator-reported acute exacerbation, and 2) those who did not. Between-groups comparisons were made using two-group Satterthwaite t-tests.

### Analysis 3: effect of nintedanib on HRQoL and symptoms in patients stratified into less-advanced or advanced disease subgroups

The objective of this analysis was to compare changes in HRQoL (SGRQ total, symptoms, activity, and impacts; EQ-5D VAS) and symptoms (UCSD-SOBQ and CASA-Q) between patients on nintedanib versus placebo, within subgroups defined as “less advanced” or “advanced” IPF using baseline measures of disease severity, including GAP (Gender, Age, Physiology) stage, FVC % predicted, DL_CO_ % predicted, CPI (composite physiologic index), and SGRQ total score (Table 1) [11, 17, 19]. Within-subgroup median treatment differences between nintedanib and placebo were calculated using the Hodges–Lehmann estimator, and statistical significance was determined using the Wilcoxon two-sample test.

**Table 1:** Classification of subgroups defined by clinical and physiologic indicators of less-advanced disease or advanced disease at baseline

Clinical measure	Subgroup with less-advanced disease	Subgroup with advanced disease
GAP stage	I	II/III
% predicted FVC, %	> 80	≤ 80
% predicted DL_CO_, %	> 40	≤ 40
CPI	≤ 45	> 45
SGRQ total score	≤ 40	> 40

*CPI* composite physiologic index, *DL*_*CO*_ diffusing capacity of the lung for carbon monoxide, *FVC* forced vital capacity, *GAP* gender, age and physiology, *SGRQ* St George’s respiratory questionnaire

## Results

### Analysis groups

The patient populations of the INPULSIS® trials have been described previously [9]. Briefly, 1061 patients were included in the two trials: 638 received nintedanib 150 mg twice daily and 423 received placebo. All patients from the INPULSIS® trials were planned to be included in these analyses; numbers of patients included in each analysis varied according to data availability.

Baseline demographic characteristics were generally similar across analysis subgroups (Tables 2, 3 and 4). Patients in different categories of FVC decline over 52 weeks had similar pulmonary function parameters at baseline (Table 2). Patients who had experienced ≥ 1 acute exacerbation (Table 3) typically had worse pulmonary function parameters at baseline than those without an acute exacerbation.

**Table 2:** Baseline characteristics of patients included in the INPULSIS® pooled data set, by category of decline in FVC % predicted (analysis 1)

	≤ 5% decline in FVC (*n* = 502)	> 5 to ≤ 10% decline in FVC (*n* = 201)	> 10% decline in FVC (*n* = 161)
Women, *n* (%)	104 (20.7)	33 (16.4)	45 (28.0)
Age, years	66.9 (8.2)	65.4 (7.5)	66.4 (7.7)
Time since IPF diagnosis, years	1.7 (1.4)	1.6 (1.3)	1.5 (1.2)
Ethnicity, *n* (%)			
White	287 (57.2)	119 (59.2)	90 (55.9)
Black	2 (0.4)	0 (0)	0 (0)
Asian	149 (29.7)	56 (27.9)	50 (31.1)
Missing	64 (12.7)	26 (12.9)	21 (13.0)
BMI, kg/m^2^	28.3 (4.4)	28.1 (4.1)	27.0 (4.9)
Smoking history, *n* (%)			
Non-smoker	128 (25.5)	64 (31.8)	50 (31.1)
Ex-smoker	345 (68.7)	132 (65.7)	108 (67.1)
Current smoker	29 (5.8)	5 (2.5)	3 (1.9)
Comorbidities, *n* (%)			
PH	17 (3.4)	6 (3.0)	3 (1.9)
COPD	15 (3.0)	4 (2.0)	2 (1.2)
Lung cancer^a^	3 (0.6)	1 (0.5)	0 (0)
GERD	122 (24.3)	43 (21.4)	42 (26.1)
CAD	48 (9.6)	9 (4.5)	12 (7.5)
FVC, % predicted	79.2 (17.3)	82.0 (18.1)	81.2 (18.0)
FEV1/FVC ratio	81.3 (5.6)	81.2 (5.8)	82.4 (6.7)
DL_CO_, % predicted	48.1 (13.1)	48.5 (12.4)	46.8 (13.1)

Data are shown in mean (standard deviation), unless otherwise indicated. ^a^Lung cancer includes malignant lung neoplasm, squamous cell carcinoma of lung, non-small-cell lung cancer and metastatic lung adenocarcinoma

*BMI* body mass index, *CAD* coronary artery disease, *COPD* chronic obstructive pulmonary disease, *DL*_*CO*_ diffusing capacity of the lungs for carbon monoxide, *FEV*_*1*_ forced expiratory volume in 1 s, *FVC* forced vital capacity, *GERD* gastroesophageal reflux disease, *IPF* idiopathic pulmonary fibrosis, *PH* pulmonary hypertension

**Table 3:** Baseline characteristics of patients included in the INPULSIS® pooled data set, experiencing no acute exacerbations or experiencing ≥ 1 acute exacerbation (analysis 2)

	No acute exacerbations (*n* = 998)	≥ 1 acute exacerbation (*n* = 63)
Women, *n* (%)	209 (20.9)	11 (17.5)
Age, years	66.6 (8.0)	69.3 (7.5)
Time since IPF diagnosis, years	1.6 (1.3)	1.8 (1.4)
Ethnicity, *n* (%)		
White	578 (57.9)	30 (47.6)
Black	2 (0.2)	0 (0)
Asian	304 (30.5)	18 (28.6)
Missing	114 (11.4)	15 (23.8)
BMI, kg/m^2^	28.0 (4.6)	27.1 (4.1)
Smoking history, *n* (%)		
Non-smoker	281 (28.2)	15 (23.8)
Ex-smoker	670 (67.1)	48 (76.2)
Current smoker	47 (4.7)	0 (0)
Comorbidities, *n* (%)		
PH	30 (3.0)	0 (0)
COPD	25 (2.5)	2 (3.2)
Lung cancer^a^	4 (0.4)	0 (0)
GERD	231 (23.1)	17 (27.0)
CAD	85 (8.5)	3 (4.8)
FVC, % predicted	80.2 (17.8)	69.4 (15.0)
FEV_1_/FVC ratio	81.5 (5.9)	84.2 (5.5)
DL_CO_, % predicted	47.5 (13.3)	42.6 (15.2)

Data are shown in mean (standard deviation), unless otherwise indicated. ^a^Lung cancer includes malignant lung neoplasm, squamous cell carcinoma of lung, non-small-cell lung cancer and metastatic lung adenocarcinoma

*BMI* body mass index, *CAD* coronary artery disease, *COPD* chronic obstructive pulmonary disease, *DL*_*CO*_ diffusing capacity of the lungs for carbon monoxide, *FEV*_*1*_ forced expiratory volume in 1 s, *FVC* forced vital capacity, *GERD* gastroesophageal reflux disease, *IPF* idiopathic pulmonary fibrosis, *PH* pulmonary hypertension

**Table 4:** Baseline characteristics of patients included in the INPULSIS® pooled data set, by stratification subgroup (analysis 3)

	Entire cohort (*N* = 1061)	GAP		FVC		DL_CO_		CPI		SGRQ	
		I (*n* = 500)	II/III (*n* = 560)	> 80% (*n* = 485)	≤ 80% (*n* = 576)	> 40% (*n* = 709)	≤ 40% (*n* = 351)	≤ 45 (*n* = 462)	> 45 (*n* = 598)	≤ 40 (*n* = 555)	> 40 (*n* = 488)
Women, *n* (%)	220 (20.7)	160 (32.0)	59 (10.5)	119 (24.5)	101 (17.5)	136 (19.2)	83 (23.6)	102 (22.1)	117 (19.6)	99 (17.8)	112 (23.0)
Age, years	66.8 (8.0)	63.0 (8.1)	70.1 (6.3)	67.8 (7.7)	65.9 (8.2)	66.4 (8.0)	67.4 (8.1)	66.1 (8.0)	67.2 (8.1)	66.1 (7.9)	67.4 (8.2)
Time since IPF diagnosis, years	1.6 (1.3)	1.6 (1.3)	1.7 (1.3)	1.6 (1.3)	1.7 (1.3)	1.6 (1.3)	1.7 (1.4)	1.6 (1.3)	1.6 (1.3)	1.5 (1.3)	1.7 (1.3)
Ethnicity, *n* (%)											
White	608 (57.3)	283 (56.6)	324 (57.9)	263 (54.2)	345 (59.9)	426 (60.1)	181 (51.6)	273 (59.1)	334 (55.9)	297 (53.5)	301 (61.7)
Black	2 (0.2)	0 (0)	2 (0.4)	0 (0)	2 (0.3)	2 (0.3)	0 (0)	0 (0)	2 (0.3)	1 (0.2)	1 (0.2)
Asian	322 (30.3)	154 (30.8)	168 (30.0)	154 (31.8)	168 (29.2)	198 (27.9)	124 (35.3)	134 (29.0)	188 (31.4)	188 (33.9)	133 (27.3)
Missing	129 (12.2)	63 (12.6)	66 (11.8)	68 (14.0)	61 (10.6)	83 (11.7)	46 (13.1)	55 (11.9)	74 (12.4)	69 (12.4)	53 (10.9)
BMI, kg/m^2^	27.9 (4.6)	28.2 (4.5)	27.6 (4.6)	27.6 (4.1)	28.1 (5.0)	28.2 (4.5)	27.3 (4.6)	28.1 (4.2)	27.8 (4.8)	27.3 (3.9)	28.6 (5.1)
Smoking history, *n* (%)											
Non-smoker	296 (27.9)	161 (32.2)	134 (23.9)	127 (26.2)	169 (29.3)	192 (27.1)	103 (29.3)	130 (28.1)	165 (27.6)	145 (26.1)	146 (29.9)
Ex-smoker	718 (67.7)	308 (61.6)	410 (73.2)	325 (67.0)	393 (68.2)	480 (67.7)	238 (67.8)	302 (65.4)	416 (69.6)	383 (69.0)	322 (66.0)
Current smoker	47 (4.4)	31 (6.2)	16 (2.9)	33 (6.8)	14 (2.4)	37 (5.2)	10 (2.8)	30 (6.5)	17 (2.8)	27 (4.9)	20 (4.1)
Comorbidities, *n* (%)											
PH	30 (2.8)	7 (1.4)	23 (4.1)	15 (3.1)	15 (2.6)	17 (2.4)	13 (3.7)	10 (2.2)	20 (3.3)	10 (1.8)	20 (4.1)
COPD	27 (2.5)	9 (1.8)	18 (3.2)	11 (2.3)	16 (2.8)	17 (2.4)	10 (2.8)	10 (2.2)	17 (2.8)	9 (1.6)	18 (3.7)
Lung cancer^a^	4 (0.4)	2 (0.4)	2 (0.4)	3 (0.6)	1 (0.2)	3 (0.3)	1 (0.3)	2 (0.4)	2 (0.4)	1 (0.2)	3 (0.6)
GERD	248 (23.4)	113 (22.6)	135 (24.1)	115 (23.7)	133 (23.1)	178 (25.1)	70 (19.9)	118 (25.5)	130 (21.7)	108 (19.5)	133 (27.3)
CAD	88 (8.3)	26 (5.2)	62 (11.1)	36 (7.4)	52 (9.0)	54 (7.6)	34 (9.7)	29 (6.3)	59 (9.9)	44 (7.9)	44 (9.0)
FVC, % predicted	79.6 (17.8)	86.4 (17.9)	73.5 (15.3)	95.2 (12.9)	66.4 (8.0)	83.3 (18.0)	72.1 (14.9)	90.4 (17.2)	71.2 (13.2)	83.1 (18.1)	75.4 (16.7)
FEV_1_/FVC ratio	81.7 (5.9)	81.3 (5.8)	82.0 (6.0)	79.8 (5.8)	83.2 (5.5)	81.0 (5.9)	83.1 (5.7)	79.5 (5.8)	83.3 (5.4)	81.0 (5.7)	82.4 (6.1)
DL_CO_, % predicted	47.2 (13.5)^†^	53.8 (13.9)	41.4 (9.9)	51.3 (12.9)	43.8 (13.0)	53.8 (11.4)	33.9 (4.6)	58.2 (11.7)	38.8 (7.2)	49.5 (12.4)	44.8 (14.2)

Data are shown in mean (standard deviation), unless otherwise indicated

*BMI* body mass index, *CAD* coronary artery disease, *COPD* chronic obstructive pulmonary disease, *CPI* composite physiologic index, *DL*_*CO*_ diffusing capacity of the lung for carbon monoxide, *FEV*_*1*_ forced expiratory volume in 1 s, *FVC* forced vital capacity, *GAP* gender, age and physiology, *GERD* gastroesophageal reflux disease, *IPF* idiopathic pulmonary fibrosis, *PH* pulmonary hypertension, *SGRQ* St George’s respiratory questionnaire

^a^Lung cancer includes malignant lung neoplasm, squamous cell carcinoma of lung, non-small-cell lung cancer and metastatic lung adenocarcinoma; ^†^Number of patients is 1060

### Impact of decline in FVC on HRQoL and symptoms

The majority of patients had ≤ 5% decline in FVC % predicted at week 52; this stratum exhibited only small changes in HRQoL and symptoms. Compared to patients in this stratum, the stratum with > 5 to ≤ 10% decline in FVC % predicted had a significantly greater degree of worsening in symptoms and HRQoL across all PROs, while patients in the stratum with > 10% decline in FVC % predicted had even greater worsening in HRQoL and symptoms (Table 5). In patients with a decline in FVC % predicted of ≤ 5%, there was no difference between the nintedanib and placebo groups in changes in HRQoL or symptoms (see Additional file 1: Table S1).

**Table 5:** Mean changes from baseline to week 52 in all PROs, reported by patients with ≤ 5%, > 5 to ≤ 10%, or > 10% decline in FVC % predicted over the study period (analysis 1)

	Mean change from baseline to week 52		
	≤ 5% decline in FVC	> 5 to ≤ 10% decline in FVC	> 10% decline in FVC
SGRQ total score	−0.18 (*n* = 477)	4.84** (*n* = 194)	13.10***^, ‡^ (*n* = 152)
SGRQ symptom score	−2.39 (*n* = 492)	7.46*** (*n* = 198)	9.40*** (*n* = 155)
SGRQ activity score	0.78 (*n* = 486)	5.75** (*n* = 196)	15.58***^, ‡^ (*n* = 154)
SGRQ impacts score	0.28 (*n =* 482)	4.02* (*n* = 198)	13.15***^, ‡^ (*n* = 153)
UCSD-SOBQ	3.02 (*n* = 441)	7.65** (*n* = 176)	15.85***^, †^ (*n* = 132)
CASA-Q cough symptom score^a^	2.47 (*n* = 495)	−3.90** (*n* = 201)	−5.27*** (*n* = 155)
CASA-Q cough impact score^a^	1.38 (*n* = 495)	−6.48*** (*n* = 200)	−8.59*** (*n* = 156)
EQ-5D VAS^a^	−0.69 (*n* = 490)	−4.78** (*n* = 197)	−10.83***^, †^ (*n* = 151)

*CASA-Q* cough and sputum assessment questionnaire (symptom and impact score), *EQ-5D VAS* EuroQoL 5-dimensional quality of life questionnaire visual analog scale, *FVC* forced vital capacity, *PRO* patient-reported outcome, *SGRQ* St George’s respiratory questionnaire (total, symptoms, activity and impacts score), *UCSD-SOBQ* University of California San Diego shortness of breath questionnaire

*P < 0.05; **P < 0.01; and ***P < 0.0001 vs the group with ≤ 5% decline in FVC

^†^*P* < 0.01 and ^‡^*P* < 0.0001 vs the group with > 5 to ≤ 10% decline in FVC

^a^Decrease in score indicates worsening health

Sensitivity analyses, in which missing values were imputed, showed similar results to the main analyses (sensitivity analyses in patients with ≤ 5% decline in FVC % predicted at week 52 are presented in Additional file 1: Tables S2 [LOCF] and S3 [WOCF]).

### Impact on HRQoL and symptoms of acute exacerbations

Patients who did not experience an acute exacerbation during the INPULSIS® studies showed some deterioration in HRQoL and symptoms. Compared with patients who had no acute exacerbations, patients with ≥1 acute exacerbation experienced numerically greater worsening across all PROs except the CASA-Q symptom score; these differences reached statistical significance for the SGRQ (total and impacts scores) and the UCSD-SOBQ (Table 6).

**Table 6:** PRO mean changes from baseline to week 52, reported by patients experiencing ≥ 1 acute exacerbation and those experiencing no acute exacerbations over the study period (analysis 2)

	Mean change from baseline to week 52	
	No acute exacerbations	≥ 1 acute exacerbation
SGRQ total score	3.18 (*n* = 808)	16.53** (*n* = 28)
SGRQ symptom score	1.93 (*n* = 829)	11.09 (*n* = 29)
SGRQ activity score	4.53 (*n* = 821)	12.44 (*n* = 28)
SGRQ impacts score	3.13 (*n* = 818)	21.27*** (*n* = 28)
UCSD-SOBQ	6.20 (*n* = 735)	22.00* (*n* = 23)
CASA-Q cough symptom score^a^	−0.63 (*n* = 835)	0.86 (*n* = 29)
CASA-Q cough impact score^a^	−2.37 (*n* = 835)	− 8.94 (*n* = 29)
EQ-5D VAS^a^	− 3.65 (*n* = 821)	−7.31 (*n* = 29)

*CASA-Q* cough and sputum assessment questionnaire (symptom and impact score), *EQ-5D VAS* EuroQoL 5-dimensional quality of life questionnaire visual analog scale, *FVC* forced vital capacity, *PRO* patient-reported outcome, *SGRQ* St George’s respiratory questionnaire (total, symptoms, activity and impacts score), *UCSD-SOBQ* University of California San Diego shortness of breath questionnaire

*P < 0.05;**P < 0.01; and ***P < 0.001 vs ‘no acute exacerbations’ group

^a^Decrease in score indicates worsening health

### Effect of nintedanib on HRQoL and symptoms in patients stratified into less-advanced or advanced disease subgroups

**Fig. 1:**
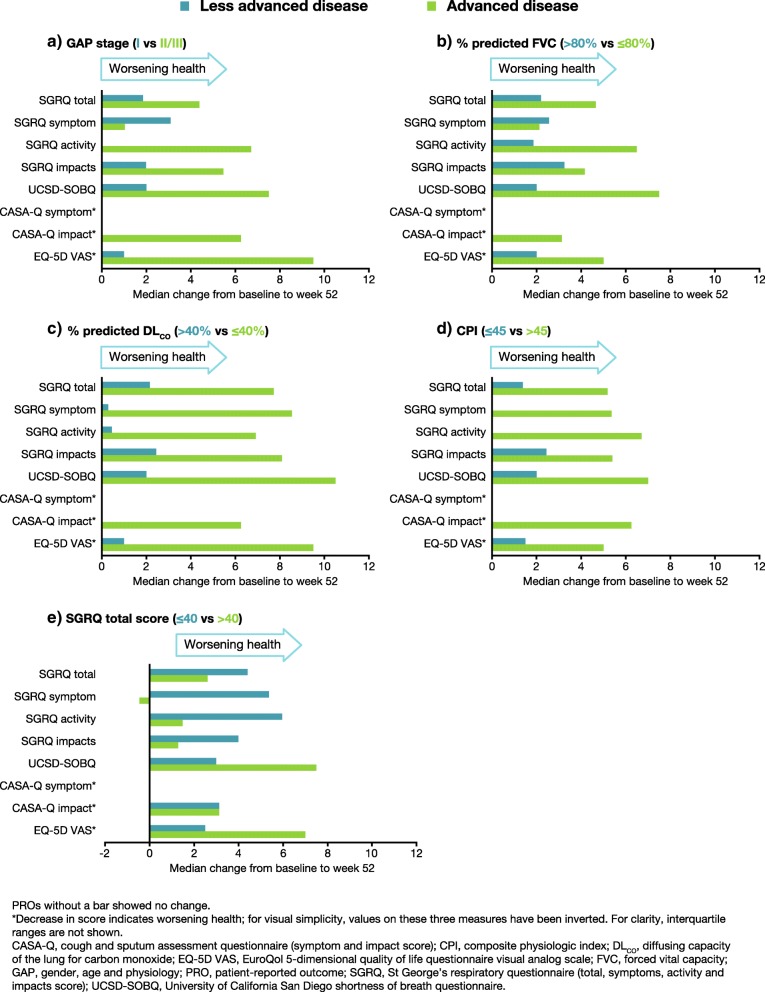
Median absolute change from baseline to week 52 across all assessed PROs in placebo-treated patients, by **a**) GAP stage, **b**) % predicted FVC, **c**) % predicted DL_CO_, **d**) CPI and **e**) SGRQ total score (analysis 3)

**Fig. 2:**
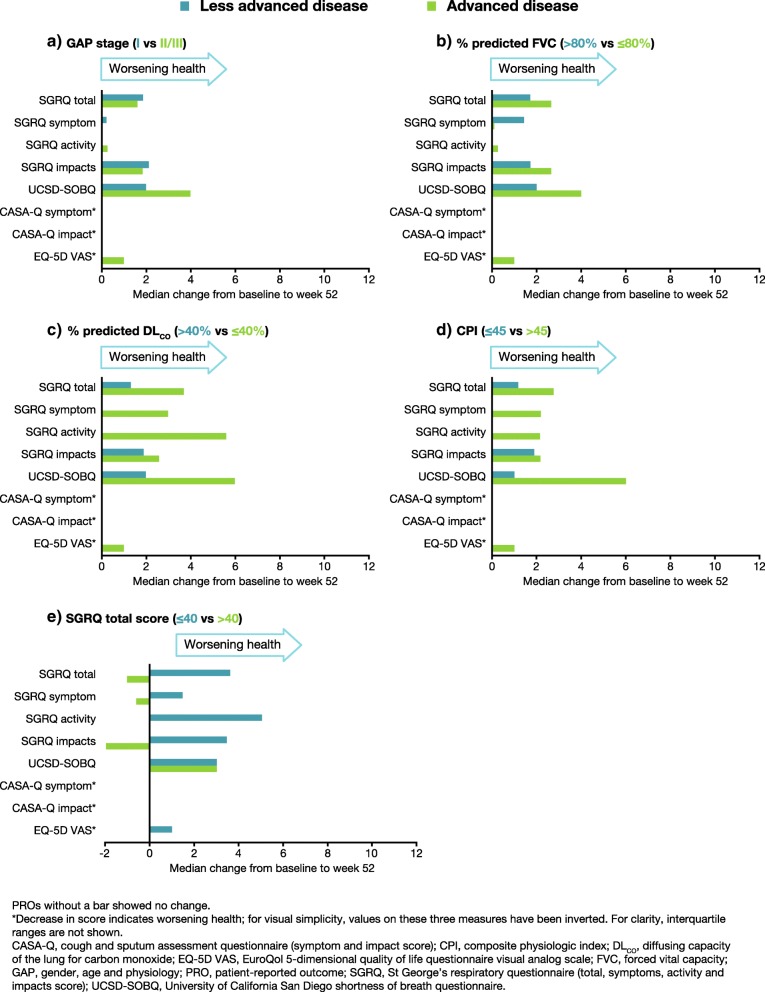
Median absolute change from baseline to week 52 across all assessed PROs in nintedanib-treated patients, by **a**) GAP stage, **b**) % predicted FVC, **c**) % predicted DL_CO_, **d**) CPI and **e**) SGRQ total score (analysis 3)

Stratification resulted in five subgroups with less-advanced disease (GAP stage I; FVC % predicted > 80%; DL_CO_ % predicted > 40%; CPI ≤ 45; SGRQ total ≤ 40) and five subgroups with advanced disease (GAP stage II or III; FVC % predicted ≤80%; DL_CO_ % predicted ≤ 40%; CPI > 45; SGRQ total > 40). At week 52, all stratification subgroups showed deterioration across almost all PRO measures. A notable exception to this was CASA-Q symptom score, for which no subgroups showed any overall change (Figs. 1 and 2).

Patients with advanced disease typically showed more deterioration in PROs than those with less-advanced disease. The exception to this pattern was patients whose SGRQ total score at baseline was > 40, who showed much less decline on all SGRQ scores (total, symptom, activity or impacts) at week 52 than patients whose SGRQ total score at baseline was ≤ 40 (Fig. 1e and Fig. 2e).

Among placebo-treated patients with advanced disease, the greatest changes from baseline were observed in scores from the UCSD-SOBQ, CASA-Q impact, and EQ-5D VAS (Fig. 1). Among nintedanib-treated patients with advanced disease, the greatest changes from baseline were observed in scores from the SGRQ (total and impacts) and UCSD-SOBQ (Fig. 2). Nintedanib-treated patients with advanced disease defined according to GAP stage or FVC % predicted showed little or no change from baseline in SGRQ symptom, SGRQ activity, CASA-Q symptom, CASA-Q impact, and EQ-5D VAS scores at week 52. Notably, nintedanib-treated patients whose SGRQ total score was > 40 at baseline showed no change or small improvements on the SGRQ (total, symptom, activity or impacts scores) at week 52 (Fig. 2e).

Overall, nintedanib-treated patients showed less decline in HRQoL measurements than placebo-treated patients (Fig. 1 versus Fig. 2). Compared with placebo-treated patients with advanced disease, nintedanib-treated patients with advanced disease showed significantly less deterioration from baseline to week 52 for several PROs. This was most apparent on the SGRQ (total and activity scores), UCSD-SOBQ and EQ-5D VAS (Table 7). The largest benefit relative to placebo was seen among nintedanib-treated patients with advanced disease (defined as GAP stage II/III, DL_CO_ ≤ 40% predicted or CPI >  45) on the SGRQ activity score. There was a numerical trend towards a reduction in deterioration from baseline with nintedanib compared with placebo for some additional PROs, including in some subgroups with less-advanced disease. However, these did not reach statistical significance.

**Table 7:**
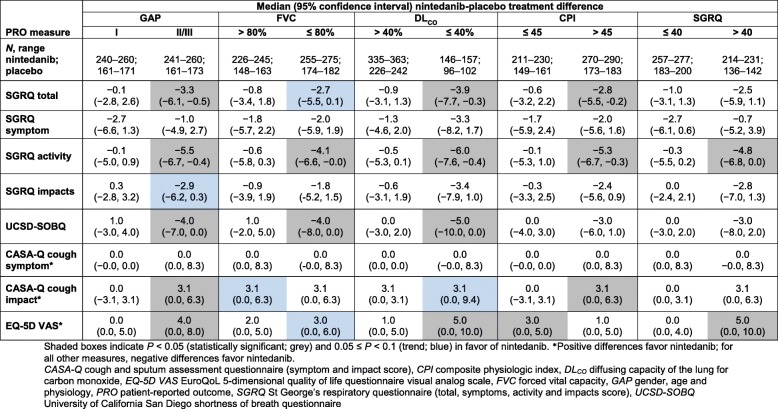
Nintedanib-placebo median treatment difference for absolute change from baseline to 52 weeks on each PRO measure, by stratification subgroup (analysis 3)

## Discussion

In patients with IPF enrolled in the INPULSIS® trials, greater categorical declines from baseline in FVC % predicted over 52 weeks were associated with worsening HRQoL and symptoms across all PRO measures. In the subgroup of patients with > 10% decline in FVC % predicted, mean change scores exceeded the MID on all domains of the SGRQ. Experiencing an acute exacerbation was associated with greater deteriorations in HRQoL and symptoms over time compared with not experiencing an acute exacerbation. In the pooled analysis of all subjects, there was no significant effect of nintedanib on HRQoL. However, subgroup analyses suggest that in patients with more advanced disease at baseline, there was significantly less deterioration in certain HRQoL domains and symptom measures among nintedanib-treated patients compared with placebo-treated patients.

The study finding that greater declines in FVC are associated with significantly greater worsening of HRQoL in patients with IPF is in agreement with previously published data and supports the validity of the instruments used. In an Australian IPF registry study of 516 patients, each 1% decline in FVC % predicted was associated with a 0.30-point increase (indicating a worsening) in total SGRQ (*P* < 0.0001) [7]. In an analysis of the German INSIGHTS-IPF registry, lower FVC % predicted, higher GAP stage and higher CPI at baseline were all associated with higher SGRQ scores [4]. At 1 year of follow-up, INSIGHTS-IPF patients with stable or improved FVC % predicted had no significant change in SGRQ total score, whereas SGRQ total scores worsened by 4 points in patients with a 0–10% decline in FVC % predicted and by 9 points in patients with a > 10% decline in FVC. Similar outcomes were seen on the EQ-5D VAS and UCSD-SOBQ [5]. The Idiopathic Pulmonary Fibrosis–Prospective Outcomes (IPF-PRO) registry is a multicenter outpatient registry of US patients with IPF. IPF-PRO collects PRO data from patients every 6 months. We eagerly await HRQoL data from this registry; they are expected to be presented in 2020.

Acute exacerbations have been defined as an acute, clinically significant respiratory deterioration characterized by evidence of new widespread alveolar abnormality. In this study, a clear numerical decline in HRQoL and symptoms was observed among patients experiencing acute exacerbations compared with those who did not. Similar findings have been reported by Collard et al. in a 2013 retrospective review of subjects enrolled in the Sildenafil Trial of Exercise Performance in IPF (STEP-IPF) who experienced a respiratory serious adverse event during the trial. Patients with any acute worsening showed significantly higher SGRQ and UCSD-SOBQ scores than those experiencing no acute worsening [20]. Similarly, a 2017 Japanese study showed that acute exacerbations resulted in a substantial decrease in activities of daily living (ADL), and persistent hypoxemia in these patients was significantly associated with reduced ADL [21].

Some results from the stratified analyses suggest that in patients with advanced IPF at baseline, compared with placebo, treatment with nintedanib is associated with less deterioration in some HRQoL domains and symptoms. Patients with advanced IPF have the most impaired HRQoL [4], as well as the poorest survival outcomes [19], likely due to disease progression. Our results suggest that, at least among patients with the most advanced IPF, reducing the rate of FVC decline reduces impairment in HRQoL and symptoms. This effect was not apparent in patients with less-advanced disease. A possible explanation is that the HRQoL measures used in this study may have a greater sensitivity for change in advanced disease and not capture more subtle changes in patients with less-advanced disease. Qualitative interviews of patients may add valuable information as to whether this is the case, and why.

In patients with IPF, adverse effects of pharmacologic therapy could impair HRQoL. However, among patients with physiologically stable IPF, the absence of a decline in HRQoL would suggest that treatment with nintedanib does not negatively impact HRQoL. Pirfenidone failed to show a significant benefit compared with placebo in the improvement of dyspnea (measured by UCSD-SOBQ) in Phase III trials [22, 23]. Nevertheless, *post hoc* pooled analyses of the full Phase III data set revealed significantly less deterioration over time in UCSD-SOBQ with pirfenidone compared with placebo. A significant treatment difference of approximately 4 points on the UCSD-SOBQ was seen at 12 months in patients with GAP stage II/III and/or baseline FVC < 80% predicted [24], almost identical to our findings with nintedanib. A median treatment difference of 8 points was reported in patients defined as having more advanced lung function impairment (FVC < 50% predicted and/or DL_CO_ < 35% predicted) [25]. An observational study in patients with daily IPF-related cough suggested that pirfenidone treatment may improve cough-related quality of life (QoL) compared to before treatment, although disease-specific QoL (measured by King's Brief Interstitial Lung disease [K-BILD]) was unchanged [26]. The AmbOx trial compared the effects of ambulatory oxygen with no oxygen on HRQoL in patients with interstitial lung disease with isolated exertional hypoxia. Compared with no oxygen, ambulatory oxygen was associated with significant improvements in K-BILD questionnaire total score and the breathlessness and activity subdomains [27].

A key strength of our study is the inclusion of a large and well-defined cohort of patients with IPF. The large numbers support the robustness of our findings. However, several study limitations should be noted. Although a broad range of patients were included in the INPULSIS® trials, patients with severe physiologic impairment (FVC < 50% predicted, DL_CO_ < 30% predicted) were excluded. The analyses were not prespecified, and thus all results should be interpreted with caution. Outcomes in subgroups based on decline in FVC % predicted or occurrence of acute exacerbations over 52 weeks were compared using post-baseline data. Only patients with available data for each PRO were included in analyses, and thus bias could have been introduced if missingness were non-ignorable. However, sensitivity analyses support the main findings. Investigator-assigned acute exacerbations may differ from adjudicated acute exacerbations. Additionally, there was substantial disparity in patient numbers in the acute exacerbations analysis between those who experienced ≥1 acute exacerbation and those who did not, due to the low number of patients with acute exacerbations and their high dropout rate. The timing of acute exacerbation relative to HRQoL measurement may also have been a factor, as a recent exacerbation may appear to have a greater impact on HRQoL. It should also be noted that the key HRQoL measure in this study, the SGRQ, was developed in patients with asthma or chronic obstructive pulmonary disease (COPD), not IPF [12]. As such, it may not provide the most accurate representation of HRQoL in this disease. Indeed, in most of the advanced disease subgroups, observed changes in SGRQ scores among placebo-treated patients did not generally exceed reported MIDs. The CASA-Q was similarly developed in patients with COPD, and the lack of change in symptom scores over 52 weeks suggests this measure may not be not as relevant in patients with IPF, in whom cough is generally dry [15]. For future research, the K-BILD questionnaire may be more appropriate considering its correlation with the EQ-5D and capacity to record disease-specific aspects of IPF [16].

HRQoL and symptomatic treatment benefits may be hard to detect in patients with less-advanced disease, because the relationship between lung function and HRQoL is not linear and may well depend on other factors such as loss of physiologic reserve and development of pulmonary hypertension. Additionally, PRO measures have a higher sensitivity for change in advanced disease. Analyses such as these can provide insights into which PROs detect changes in clinical and physiologic function in IPF efficiently and accurately, and help to improve our understanding of the relationship between HRQoL and symptom burden in IPF.

## Conclusions

These analyses provide much-needed new data on the potential value of HRQoL and symptom scores in clinical trials of patients with IPF. In patients with advanced IPF, compared with placebo, treatment with nintedanib was associated with less deterioration in HRQoL. These findings suggest that, by reducing the annual rate of FVC decline, nintedanib may have similar beneficial effects on outcomes that are equally important to patients.

## Supplementary information

**Additional file 1: Table S1.** Mean and median changes from baseline to week 52 and median differences between nintedanib and placebo in PROs reported by patients with ≤ 5% decline in FVC % predicted over the study period (analysis 1). **Table S2.** Mean and median changes from baseline to week 52 and median differences between nintedanib and placebo in PROs reported by patients with ≤ 5% decline in FVC % predicted over the study period (LOCF sensitivity analysis). **Table S3.** Mean and median changes from baseline to week 52 and median differences between nintedanib and placebo in PROs reported by patients with ≤ 5% decline in FVC % predicted over the study period (WOCF sensitivity analysis).

## Data Availability

The data that support the findings of this study are available from https://trials.boehringer-ingelheim.com/ but restrictions apply to the availability of these data, which were used under license for the current study, and so are not publicly available. Data are however available from the authors upon reasonable request and with permission of Boehringer Ingelheim.

## Abbreviations

ADL: Activities of daily living
BMI: Body mass index
CAD: Coronary artery disease
CASA-Q: Cough and Sputum Assessment Questionnaire
COPD: Chronic obstructive pulmonary disease
CPI: Composite physiologic index
DL_CO_: Diffusing capacity of the lung for carbon monoxide
EQ-5D: EuroQoL 5-dimensional quality of life questionnaire
FVC: Forced vital capacity
GAP: Gender, age and physiology
GERD: Gastroesophageal reflux disease
HRQoL: Health-related quality of life
IPF: Idiopathic pulmonary fibrosis
IPF-PRO: IPF-Prospective Outcomes Registry
K-BILD: King’s Brief Interstitial Lung Disease (questionnaire)
LOCF: Last observation carried forward
MID: Minimally important difference
PH: Pulmonary hypertension
PRO: Patient-reported outcome
QoL: Quality of life
SGRQ: St George’s respiratory questionnaire
STEP-IPF: Sildenafil Trial of Exercise Performance in IPF
UCSD-SOBQ: University of California San Diego shortness of breath questionnaire
VAS: Visual analog scale
WOCF: Worst observation carried forward
