# Supplementary material for: Health-related quality of life and symptoms in patients with IPF treated with nintedanib: analyses of patient-reported outcomes from the INPULSIS® trials
Source: Respir Res. 2020 Jan 30;21:36. doi: 10.1186/s12931-020-1298-1 (PMC6990488; doi:10.1186/s12931-020-1298-1)
Supplement: Supplementary file 1 — Additional file 1: Table S1. Mean and median changes from baseline to week 52 and median differences between nintedanib and placebo in PROs reported by patients with ≤ 5% decline in FVC % predicted over the study period (analysis 1). Table S2. Mean and median changes from baseline to week 52 and median differences between nintedanib and placebo in PROs reported by patients with ≤ 5% decline in FVC % predicted over the study period (LOCF sensitivity analysis). Table S3. Mean and median changes from baseline to week 52 and median differences between nintedanib and placebo in PROs reported by patients with ≤ 5% decline in FVC % predicted over the study period (WOCF sensitivity analysis). [file 12931_2020_1298_MOESM1_ESM.pdf]

Health-related quality of life and symptoms in patients with IPF treated with nintedanib: analyses of patient-reported outcomes from the INPULSIS® trials

Michael Kreuter, Wim A Wuyts, Marlies Wijsenbeek, Sabrina Bajwah, Toby M Maher, Susanne Stowasser, Natalia Male, Wibke Stansen, Nils Schoof, Leticia Orsatti and Jeffrey Swigris

Supplementary material

A separate analysis comparing nintedanib- and placebo-treated patients who showed a ≤ 5% decline in FVC % predicted was performed. For this comparison, median difference in change from baseline to week 52 between nintedanib and placebo for each PRO was calculated using the Hodges–Lehmann estimator, and statistical significance was determined using the Wilcoxon two-sample test.

Table S1. Mean and median changes from baseline to week 52 and median differences between nintedanib and placebo in PROs reported by patients with ≤ 5% decline in FVC % predicted over the study period (analysis 1)

	**Mean change from baseline to week 52**		**Median change from baseline to week 52**		**Median difference in change from baseline (nintedanib vs placebo)**	***P* value**
	**Placebo**	**Nintedanib**	**Placebo**	**Nintedanib**		
**SGRQ total score**	0.07 (*n* = 162)	−0.31 (*n* = 315)	0.08 (*n* = 162)	−0.91 (*n* = 315)	−0.66 (−3.06, 1.82)	0.602
**SGRQ symptom score**	−2.68 (*n* = 163)	−2.25 (*n* = 329)	−2.80 (*n* = 163)	−0.52 (*n* = 329)	0.65 (−2.65, 4.05)	0.706
**SGRQ activity score**	0.76 (*n* = 163)	0.78 (*n* = 323)	0.00 (*n* = 163)	0.00 (*n* = 323)	−0.12 (−3.66, 0.56)	0.436
**SGRQ impacts score**	0.99 (*n* = 162)	−0.09 (*n* = 320)	0.46 (*n* = 162)	0.00 (*n* = 320)	−1.28 (−3.98, 1.52)	0.386
**UCSD-SOBQ**	3.11 (*n* = 145)	2.97 (*n* = 296)	2.00 (*n* = 145)	1.00 (*n* = 296)	−1.00 (−4.00, 2.00)	0.601
**CASA-Q cough symptom score***	2.15 (*n* = 163)	2.64 (*n* = 332)	0.00 (*n* = 163)	0.00 (*n* = 332)	0.00 (−0.00, 0.00)	0.985
**CASA-Q cough impact score***	0.75 (*n* = 163)	1.68 (*n* = 332)	0.00 (*n* =163)	0.00 (*n* = 332)	0.00 (−3.13, 3.13)	0.596
**EQ-5D VAS***	−3.32 (*n* = 161)	0.59 (*n* = 329)	0.00 (*n* = 161)	0.00 (*n* = 329)	3.00 (0.00, 5.00)	0.040

*Decrease in score indicates worsening health; positive differences favor nintedanib (for all other measures, negative differences favor nintedanib).
CASA-Q cough and sputum assessment questionnaire (symptom and impact score), *EQ-5D VAS* EuroQoL 5-dimensional quality of life questionnaire visual analog scale, *FVC* forced vital capacity, *PRO* patient-reported outcome, *SGRQ* St George's respiratory questionnaire (total, symptoms, activity and impacts score), *UCSD-SOBQ* University of California San Diego shortness of breath questionnaire

Table S2. Mean and median changes from baseline to week 52 and median differences between nintedanib and placebo in PROs reported by patients with ≤ 5% decline in FVC % predicted over the study period (LOCF sensitivity analysis)

	**Mean change from baseline to week 52**		**Median change from baseline to week 52**		**Median difference in change from baseline (nintedanib vs placebo)**	***P* value**
	**Placebo**	**Nintedanib**	**Placebo**	**Nintedanib**		
**SGRQ total score**	−0.10 (*n* = 164)	−0.01 (*n* = 321)	0.02 (*n* = 164)	−0.78 (*n* = 321)	−0.25 (−2.66, 2.25)	0.843
**SGRQ symptom score**	−3.02 (*n* = 165)	−1.77 (*n* = 335)	−3.01 (*n* = 165)	−0.30 (*n* = 335)	1.38 (−2.03, 4.73)	0.439
**SGRQ activity score**	0.76 (*n* = 165)	0.95 (*n* = 329)	0.00 (*n* = 165)	0.00 (*n* = 329)	−0.07 (−3.13, 0.69)	0.503
**SGRQ impacts score**	0.79 (*n* = 164)	0.24 (*n* = 326)	0.45 (*n* = 164)	0.00 (*n* = 326)	−0.78 (−3.56, 1.90)	0.583
**UCSD-SOBQ**	3.30 (*n* = 147)	3.42 (*n* = 300)	2.00 (*n* = 147)	1.00 (*n* = 300)	−1.00 (−4.00, 2.00)	0.648
**CASA-Q cough symptom score***	2.27 (*n* = 165)	2.34 (*n* = 338)	0.00 (*n* = 165)	0.00 (*n* = 338)	0.00 (−0.00, 0.00)	0.854
**CASA-Q cough impact score***	0.83 (*n* = 165)	1.22 (*n* = 338)	0.00 (*n* = 165)	0.00 (*n* = 338)	0.00 (−3.13, 3.13)	0.733
**EQ-5D VAS***	−3.36 (*n* = 163)	0.33 (*n* = 334)	0.00 (*n* = 163)	0.00 (*n* = 334)	3.00 (0.00, 5.00)	0.048

*Decrease in score indicates worsening health; positive differences favor nintedanib (for all other measures, negative differences favor nintedanib).
CASA-Q cough and sputum assessment questionnaire (symptom and impact score), *EQ-5D VAS* EuroQoL 5-dimensional quality of life questionnaire visual analog scale, *FVC* forced vital capacity, *LOCF* last observation carried forward, *PRO* patient-reported outcome, *SGRQ* St George’s respiratory questionnaire (total, symptoms, activity and impacts score), *UCSD-SOBQ* University of California San Diego shortness of breath questionnaire

Table S3. Mean and median changes from baseline to week 52 and median differences between nintedanib and placebo in PROs reported by patients with ≤ 5% decline in FVC % predicted over the study period (WOCF sensitivity analysis)

	**Mean change from baseline to week 52**		**Median change from baseline to week 52**		**Median difference in change from baseline (nintedanib vs placebo)**	***P* value**
	**Placebo**	**Nintedanib**	**Placebo**	**Nintedanib**		
**SGRQ total score**	−0.10 (*n* = 164)	−0.13 (*n* = 319)	0.02 (*n* = 164)	−0.86 (*n* = 319)	−0.33 (−2.73, 2.18)	0.799
**SGRQ symptom score**	−3.02 (*n* = 165)	−1.93 (*n* = 333)	−3.01 (*n* = 165)	−0.33 (*n* = 333)	1.25 (−2.13, 4.61)	0.479
**SGRQ activity score**	0.76 (*n* = 165)	0.94 (*n* = 327)	0.00 (*n* = 165)	0.00 (*n* = 327)	−0.08 (−3.13, 0.69)	0.497
**SGRQ impacts score**	0.79 (*n* = 164)	0.06 (*n* = 324)	0.45 (*n* = 164)	0.00 (*n* = 324)	−0.90 (−3.65, 1.82)	0.528
**UCSD-SOBQ**	3.30 (*n* = 147)	3.16 ((*n* = 299)	2.00 (*n* = 147)	1.00 (*n* = 299)	−1.00 (−4.00, 2.00)	0.607
**CASA-Q cough symptom score***	2.27 (*n* = 165)	2.48 (*n* = 336)	0.00 (*n* = 165)	0.00 (*n* = 336)	0.00 (−0.00, 0.00)	0.897
**CASA-Q cough impact score***	0.83 (*n* = 165)	1.49 (*n* = 336)	0.00 (*n* = 165)	0.00 (*n* = 336)	0.00 (−3.13, 3.13)	0.675
**EQ-5D VAS***	−3.36 (*n* = 163)	0.36 (*n* = 332)	0.00 (*n* = 163)	0.00 (*n* = 332)	3.00 (0.00, 5.00)	0.045

*Decrease in score indicates worsening health; positive differences favor nintedanib (for all other measures, negative differences favor nintedanib).
CASA-Q cough and sputum assessment questionnaire (symptom and impact score); *EQ-5D VAS* EuroQoL 5-dimensional quality of life questionnaire visual analog scale, *FVC* forced vital capacity, *PRO* patient-reported outcome, *SGRQ* St George's respiratory questionnaire (total, symptoms, activity and impacts score), *UCSD-SOBQ* University of California San Diego shortness of breath questionnaire, *WOCF* worst observation carried forward
